# A budget impact analysis of 15- or 20- valent pneumococcal conjugate vaccine use in all US adults aged 50–64 years old compared to those with high-risk conditions from US payer perspective

**DOI:** 10.1186/s12889-025-22827-9

**Published:** 2025-06-02

**Authors:** Nirma Khatri Vadlamudi, Chyongchiou J. Lin, Angela R. Wateska, Richard K. Zimmerman, Kenneth J. Smith

**Affiliations:** 1 Department of Pharmaceutical Outcomes & Policy, College of Pharmacy, University of Florida, 1889 Museum Road, Suite 6300, Gainesville, FL 32611 USA; 2 College of Nursing, The Ohio State University, Columbus, OH USA; 3 Department of Medicine, University of Pittsburgh, Pittsburgh, PA USA; 4 Department of Family Medicine, University of Pittsburgh, Pittsburgh, PA USA

**Keywords:** Pneumococcal, Budget impact analyses, Vaccine, US, Adults

## Abstract

**Background:**

In 2023, the US CDC recommended 20-valent pneumococcal conjugate vaccine (PCV20) or 15-valent pneumococcal conjugate vaccine (PCV15) followed by 23-valent pneumococcal polysaccharide vaccine (PPSV23) for all adults aged 65-years and older and those aged 19–64 years old with chronic conditions. However, there is substantial pneumococcal disease burden in healthy adults aged 50–64 years, particularly in Black adults, who are likely to benefit from vaccination. This study assesses the financial impact of introducing routine PCV15 or PCV20 in US adults aged 50–64 years.

**Objective:**

To evaluate the budget impact of introducing PCV20 or PCV15/PPSV23 use in adults aged 50–64 years old compared to vaccinating only those with high-risk conditions for pneumococcal disease.

**Methods:**

A budget impact model was developed over a 3-year time horizon to compare PCV20 versus PCV15/PPSV23 from the US payer perspective. Outcomes and costs of pneumococcal disease among US adults aged 50–64 years and those with underlying conditions were projected using a Markov decision model.

**Results:**

Incorporating either PCV20 or PCV15/PPSV23 vaccines in routine vaccination programs for adults aged 50–64 years compared to vaccinating only adults with chronic conditions had an incremental budget impact of $6.5 and $9 billion, respectively, over three years. Budgetary impact was sensitive to number of vaccine doses, cost of vaccine per dose, vaccine coverage proportion and pneumococcal treatment cost across the overall population and sub-groups. Routine vaccination of 50-64-year-old age group was more economically favorable in Black adults sub-group analyses.

**Supplementary Information:**

The online version contains supplementary material available at 10.1186/s12889-025-22827-9.

## Significance

Our study provides budgetary insights for decision-makers evaluating pneumococcal vaccination programs targeting US adults aged 50–64 years.

**Supplementary Information:**

The online version contains supplementary material available at 10.1186/s12889-025-22827-9.

## Introduction

*Streptococcus pneumoniae* causes substantial morbidity and mortality among United States (US) adults [1]. Common manifestations of pneumococcal infections are invasive pneumococcal disease (IPD) and community-acquired pneumonia (CAP) [1]. US Active Bacterial Core surveillance reports that IPD incidence in adults aged ≥ 65 years was 24 per 100,000 population in 2018/19 and those aged 50–64 years was 15.6 per 100,000 [2, 3]. An estimated 1.5 million CAP cases are reported annually in US adults [4]. During 2010–2016, the annual incidence of CAP-associated hospitalizations in the U.S. was 126–422 per 100,000 in adults under 65 years and 847–3,365 per 100,000 in those 65 years and older [5]. Given the average IPD and CAP-related length of hospital stay is 10–12 days, with 18% of cases requiring mechanical ventilation and 5% needing critical care, a significant burden is posed on the healthcare system [6, 7]. Case fatality rates from IPD and CAP during hospitalization are about 11% and 7%, and high mortality rates were seen a year after hospitalization among adults aged 50 years and older [4]. 

Black adults are disproportionately affected pneumococcal disease, due to health inequities experienced by this population from pervasive structural racism [8]. IPD rates were two times higher in Black adults compared with White adults [2]. Pneumococcal disease mortality was greater by 1.1−12% in Black adults than non-Black adults, and lengths of hospital stays were generally longer for Black adults than for non-Black adults [9]. Individuals from lower socioeconomic backgrounds and Black ethnicity are similarly at higher risk for CAP [4, 9]. Due to high disease burden and persistent health inequities, this study pays special attention to Black adults in subgroup analyses.

The use of pneumococcal conjugate vaccines, which began in 2003 in children and in 2012 in adults, led to a significant decline in overall IPD cases in the US. However, there is persistent IPD and CAP burden among adults from serotypes not covered by PCV13 [2, 3]. In 2023, two new vaccines were approved for use in adults ≥ 65 years to address non-PCV13 serotype burden: 15-valent pneumococcal conjugate vaccine (PCV15)^1^ and 20-valent pneumococcal conjugate vaccine (PCV20)^2^ [2]. While the same serotypes are covered in the older PPSV23 vaccine, its effectiveness is short-lived against IPD, and it is not as efficacious against CAP [10, 11]. In 2024, ACIP also recommended PCV21 as an option for use in adults ≥ 65 years [12]. In 2019, US adults aged ≥ 65 years had 42% PCV15 serotype IPD cases and 69% PCV20 serotype IPD cases [3]. Similarly, those adults aged 19–64 years in the US had 43% PCV15 serotype IPD cases and 71% PCV20 serotype IPD cases [3]. Among all US adults with CAP, 5% matched the serotypes in PCV15 and 8% matched the serotypes in PCV20 [13]. Therefore, PCV20 or PCV15 were recommended among adults ≥ 65 years to address the residual burden in this population [2]. Since non-PCV13 serotype burden is disproportionately high in healthy adults aged 50–64 years and in Black adults, they are likely to benefit from these vaccines.

This study assesses the financial impact of introducing PCV15 or PCV20 vaccines in the US adults aged 50–64 years. We will compare the budget impact of introducing of PCV15 or PCV20 with recommendations of pneumococcal vaccines use in adults aged 50–64 years old compared with the high-risk population from a US payer perspective.

## Methods

### Model description

**Fig. 1:**
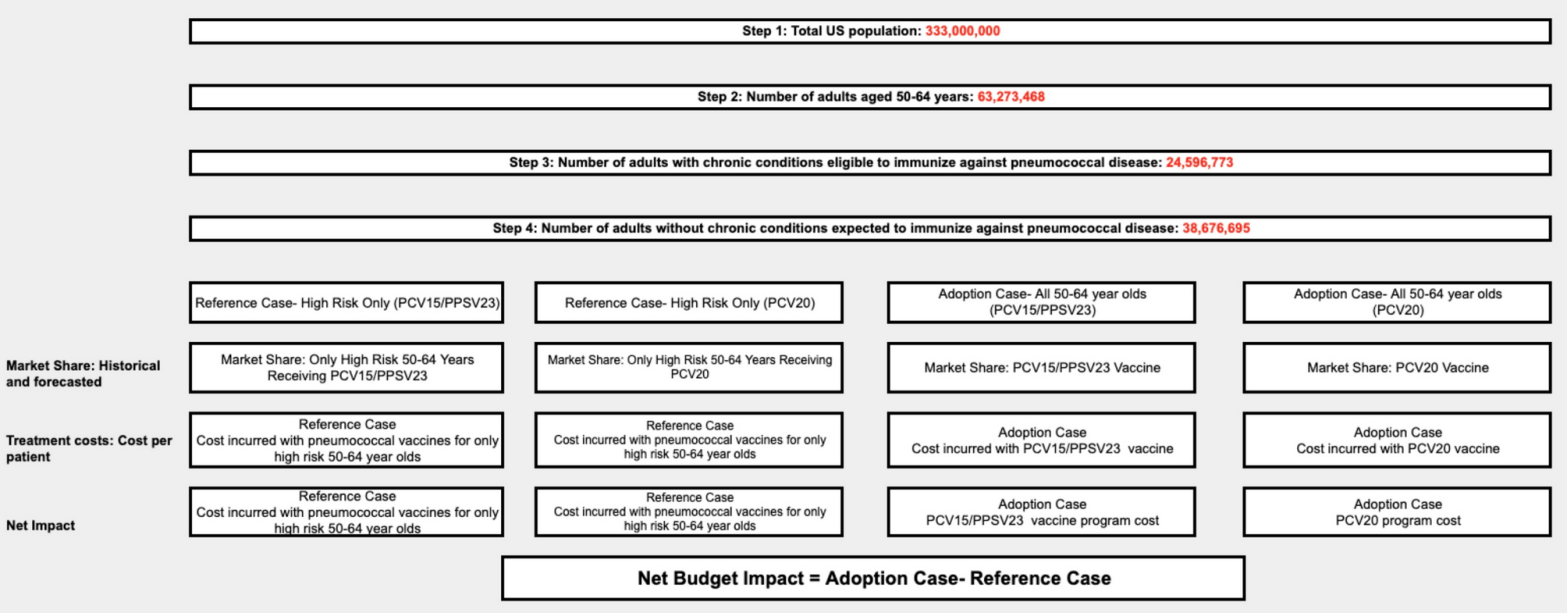
Model schematic

A deterministic model with a Markov-type process was used to depict the 3-year risk of health outcomes and associated economic costs for various pneumococcal vaccination strategies in a population of US adults aged 50–64 years (Fig. 1). For simplicity, the model population is considered stable as the number of people entering the 50-64-year age group will be the same as those leaving the age group. The population was stratified by cohort (Black or non-Black). US adults aged 50–64 years in the model were assumed to receive either PCV20 or PCV15 followed by PPSV23 (Base Case) compared with individuals with chronic conditions eligible to receive either PCV20 or PCV15 followed by PPSV23 (Reference Case) during the modelling horizon of three years. The net budget impact was calculated as the difference in expenditures between the two scenarios.

### Perspective and target audience

The analysis was conducted from the US payer perspective for funding consideration of PCV20 alone or PCV15 followed by PPSV23. Direct costs associated with vaccine and pneumococcal disease treatment were included. Indirect costs due to improved health outcomes, health insurance premiums and drug plan deductibles were excluded from the cost calculations. All costs were stated in 2022 US dollars. Discounting was not applied within the budget impact analysis (BIA) due to a short analytical time horizon [14]. 

### Time horizon

A baseline period of 12 months was described, after which two strata: Black and non-Black cohorts were forecasted for three additional years. The base year in the model was 2023, and the budget impact was projected from 2024 to 2026.

### Input data and sources

A summary of model inputs with data sources is available in Table 1. Health outcomes and associated costs are projected annually for the model population based on cohort, disease burden, mortality rates, vaccination uptake, unit costs of vaccination, and healthcare utilization. [15] Pneumococcal disease cases consisted of IPD (bacteremia, meningitis, and septicemia) and non-bacteremic pneumonia (NBP), which were stratified by outpatient and inpatient settings. Average population risk for IPD and NBP events and associated mortality varied by vaccine uptake rates over time. Therefore, a lower risk for IPD and NBP events was observed over time with increased vaccine uptake in the population as noted in Wateska et al. [15] Additionally, we adjusted for the increase in pneumococcal disease risk associated with non-vaccine serotypes and waning immunity over time since vaccination. Estimated healthcare utilization costs for IPD and NBP were based on event rates and associated unit costs stratified by the healthcare setting. Overall vaccine and vaccine administration costs were added in the year of the vaccine administration. Economic costs for health outcomes were projected for each vaccination strategy including IPD, NBP cases, and attributable deaths varying by vaccination uptake rates and healthcare resource utilization costs.

**Table 1:** Input data and sources

Main Parameter	Details	Value	Source
Population	US population	333,000,000	[16]
	All adults 50–64 years old	63,273,468	[16]
	All adults 50–64 years old with underlying conditions	24,596,773	[17]
	Black adults 50–64 years old	8,302,918	[17]
	Black adults 50–64 years old with underlying conditions	3,213,229	[17]
	Non-Black adults 50–64 years old	54,970,550	[17]
	Non-Black adults 50–64 years old with underlying conditions	21,383,544	[17]
Costs per vaccine dose	PCV20 (includes administration fees)	$278.16 (+/-50%)	[18, 20, 25]
	PCV15 (includes administration fees)	$245.25 (+/-50%)	[18, 20, 25]
	PPSV23 (includes administration fees)	$146.24 (+/-50%)	[18, 20, 25]
Vaccine coverage	For adults with chronic conditions	23%	[18, 27]
	For all adults	Mean: 10–30% Low: 5–15% High: 10–40%	Assumed
Pneumococcal treatment cost (USD 2022)			
All adults aged 50–64 years old	IPD	$28,924 (95%CI:$23139, $34709)	[18, 19, 28]
	IPD Death	$68,393 (95%CI:$54715, $82072)	[18, 19, 28]
	NBP (outpatient)	$709 (95%CI:$701, $716)	[18, 19, 28]
	NBP (inpatient)	$17,420 (95%CI:$13936, $20904)	[18, 19, 28]
	NBP death	$42,049 (95%CI:$33639, $50459)	[18, 19, 28]
Black adults aged 50–64 years old	IPD	$28,821 (95%CI:$23057, $34586)	[18, 19, 28]
	IPD Death	$60,148 (95%CI:$48118, $72177)	[18, 19, 28]
	NBP (outpatient)	$709 (95%CI:$701, $716)	[18, 19, 28]
	NBP (inpatient)	$17,301 (95%CI:$13841, $20761)	[18, 19, 28]
	NBP death	$42,281 (95%CI:$33825, $50737)	[18, 19, 28]
Non-Black adults aged 50–64 years old	IPD	$29,026 (95%CI:$23221, $34831)	[18, 19, 28]
	IPD Death	$76,638 (95%CI:$61311, $91966)	[18, 19, 28]
	NBP (outpatient)	$709 (95%CI:$701, $716)	[18, 19, 28]
	NBP (inpatient)	$17,539 (95%CI:$14031, $21047)	[18, 19, 28]
	NBP death	$41,816 (95%CI:$33453, $50180)	[18, 19, 28]

### Population

The model population size (63 million) and age distribution were based on 2022 US census data [16]. Persons in the age group of 50–64 years were stratified based on their race using the National Health Interview Survey (NHIS) and National Center for Health Statistics (NCHS) datasets [17].

### Vaccine uptake

Vaccine uptake rates for those with chronic conditions were obtained from the literature [18]. Vaccine uptake for the routine program was assumed, as listed in Table 1. Vaccine-eligible individuals were assumed to be eligible until vaccination, death, or the end of the modelling horizon.

### Base case analyses

In 2023, ACIP recommended either a single-dose PCV20 or a two-dose series PCV15 followed by PPSV23 (PCV15/PPSV23) for those individuals aged 50–64 years with chronic conditions. We proposed that the US adults aged 50–64 years should receive either a single dose PCV20 or a two-dose series PCV15 followed by PPSV23 (PCV15/PPSV23) in routine adult vaccination program. Strategy one is vaccinating the US adults aged 50–64 years of age, with alternate analyses examining the effects of vaccinating Black cohorts or non-Black cohorts. Health outcomes and economic costs were projected for the model population under PCV20 alone and PCV15, followed by PPSV23 for the US adults, including Black and non-Black adults specified in Wateska et al. [15] Analyses were conducted from the payer’s perspective. Annual economic costs were considered in the context of Medicaid as a conservative assumption of costs [18–20]. 

### Scenario analyses

One-way sensitivity analyses were conducted in which alternative input values were substituted for key model parameters such as vaccine uptake and low and high treatment costs. Inputs used in the sensitivity analyses are in Table 1.

## Results

### Vaccination coverage

A total of 63 million individuals are 50–64 years of age in the US population. Of these, about 24 million individuals have chronic conditions that are eligible to receive PCV20 or PCV15 followed by PPSV23 vaccinations. If the program was expanded to include the US adults without chronic conditions aged 50–64 years, it would add 38 million people to the pool as shown in Fig. 1. Vaccine uptake rates for 50–64 years of age with chronic conditions is around 23%. Given fewer interactions with the healthcare system for those without diagnosed chronic conditions, and assumed vaccine coverage as indicated in eTable 1, the uptake is assumed to increase from 10 to 30% over 3 three years. As such, the vaccine-eligible population will increase from 5.7 million (base year) to 17 million (year 3) over time, as depicted in supplementary eFigure 1 and eTable 2.

### Budget impact

Over a three-year time horizon, the cost per vaccine dose for the reference case was assumed to remain constant. In the adoption case, vaccine uptake increases slowly to the maximum of 53% in the overall mean coverage shown in eTable 1 and eTable2. Therefore, a gradual increase in cost is noted in eTable 3 for PCV20 alone and eTable 4 for PCV15/PPSV23. In the base case scenario, extending only the PCV20 program to the US adults aged 50–64 years old without high-risk condition would result in a net budget increase of 6.5 billion over 3 years as shown in eTable 3, and for the PCV15/PPSV23 program, the extension would cost 9 billion (eTable 4). If the PCV20 program were extended to only a cohort of Black individuals aged 50–64 years, it would cost an additional 850 million over 3 years and an incremental increase in PCV15/PPSV23 program cost will be 1.1 billion dollars (Table 2). Extending the PCV20 alone program to a non-Black 50–64-year-old cohort would be an additional 5.6 billion dollars, whereas PCV15/PPSV23 would be 7.8 billion dollars over 3 years (Table 2).

**Table 2:** Three-year budget impact for PCV20 alone and PCV15 followed by PPSV23 base case and alternate scenarios with % change from base case scenarios

	PCV20 alone	PCV15 + PPSV23
Base case	$6,454,985,653	$9,084,923,545
Alternate Scenarios:		
Low cost per vaccine dose	$3,227,492,826 (-50.0%)	$4,542,461,772 (-50.0%)
High cost per vaccine dose	$9,682,478,479 (+ 50.0%)	$13,627,385,317 (+ 50.0%)
Including mean treatment cost	$6,176,968,204 (-4.3%)	$8,761,658,114 (-3.6%)
Including low treatment cost	$6,228,618,999 (-3.5%)	$8,821,977,704 (-2.9%)
Including high treatment cost	$6,125,349,182 (-5.1%)	$8,701,373,851 (-4.2%)
Low vaccine coverage	$3,227,492,826 (-50.0%)	$4,542,461,772 (-50.0%)
High vaccine coverage	$7,530,816,595 (+ 16.7%)	$10,599,077,469 (+ 16.7%)
Low vaccine coverage and mean treatment cost	$3,062,590,973 (-52.6%)	$4,357,951,629 (-52.0%)
High vaccine coverage and mean treatment cost	$7,139,858,893 (+ 10.6%)	$10,136,971,905 (11.6%)
Subgroup: All Black adults 50–64 years old	$849,448,691 (-86.8%)	$1,195,537,345 (-86.8%)
Subgroup: All Non-Black adults 50–64 years old	$5,605,536,962 (-13.2%)	$7,889,386,199 (-13.2%)
Subgroup: All Black adults 50–64 years old and mean treatment cost	$813,857,775 (-87.4%)	$1,154,128,179 (-87.3%)
Subgroup: All Non-Black adults 50–64 years old and mean treatment cost	$5,358,677,554 (-17.0%)	$7,601,717,778 (-16.3%)

### Sensitivity analyses

**Fig. 2:**
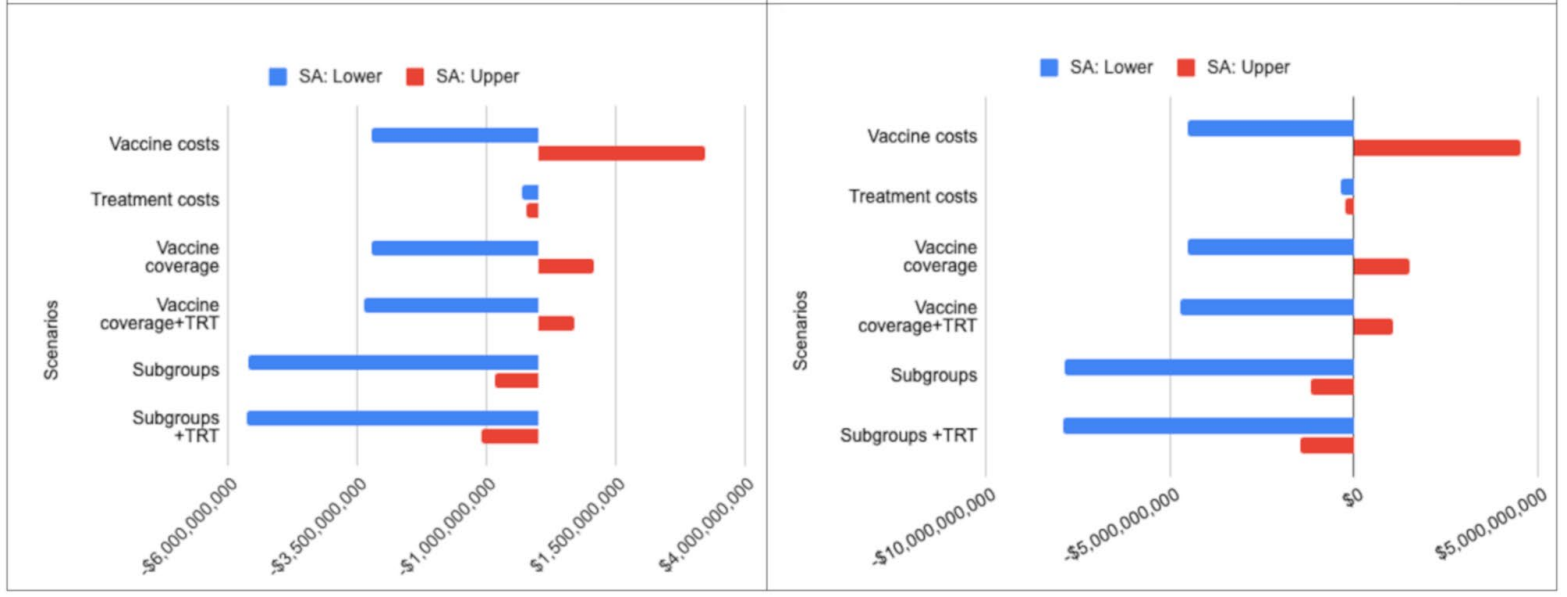
Sensitivity scenarios **A**: PCV20 **B**: PCV15/PPSV23 Abbreviation: TRT indicates mean treatment costs Vaccine costs refer cost of vaccine per dose

Sensitivity analyses include multiple scenarios, such as costs per vaccine dose, vaccination coverage, including low and high treatment costs for IPD and NBP, cohort-specific treatment costs. As seen in Fig. 2, budget impact was the lowest for the Black cohort along with treatment cost for PCV20 alone and highest for PCV15/PPSV23 for high vaccine coverage along with treatment cost compared with the base case scenarios.

## Discussion

We found the incremental budget impact of expanding the adult pneumococcal vaccination program in the US from high-risk 50-64-year-old adults to include those 50-64-year-old adults without high-risk conditions was $6.5 billion for PCV20 and $9 billion for the PCV15 followed by the PPSV23 program. In this analysis, we accounted for epidemiological changes in IPD and NBP cases induced by routine infant vaccination programs. Due to uncertainties around vaccine uptake in adults and its impact on epidemiological changes and cost of treatment, several scenarios were evaluated with all favoring one dose of PCV20 over PCV15 followed by PPSV23. Expanding the pneumococcal program to the Black adults aged 50–64 years without high-risk conditions resulted in the budget increase of 850 million.

The CDC revised adult pneumococcal vaccination recommendations from PCV13/PPSV23 in adults aged under 65 years with immunocompromising conditions, PPSV23 in those adults with other high-risk conditions or all adults aged 65 years and older to a single dose of PCV20 or a sequential dose of PCV15 followed by PPSV23 [2]. In the three cost-effectiveness models assessed by CDC and ACIP, they report that PCV20 alone for all adults aged 65 years and older ranged from cost-saving to $39,000 per quality-adjusted life-year (QALY) gained [2]. For adults aged 19–64 years with certain underlying medical conditions, CDC/ACIP reported that cost-effectiveness estimates ranged from $11,000 to $292,000 per QALY gained for PCV20 [2]. In contrast, when CDC/ACIP assessed data from two CEA models, PCV15 in series with PPSV23 for all adults aged 65 years and older, they found values ranged from cost-saving to $282,000 per QALY gained [2]. For adults aged 19–64 years with certain underlying medical conditions, costs ranged from $250,000 to $656,000 for PCV15 in series with PPSV23 [2]. This highlights that the single dose strategy was preferred for adults aged 65 years and older and those 19–64 years of age with specific underlying medical conditions.

Because social determinants of health play a key role in predisposing individuals to pneumococcal disease, CDC incorporated a health equity perspective into their decision-making and vaccine policy recommendations [2]. When the ACIP/CDC revised adult pneumococcal vaccination recommendations supported by cost-effectiveness analyses in 2022, they postulated that age-based PCV20 or PCV15 followed by PPSV23 use at age 50 years and compared to risk-based and age-based strategies were: (1) likely to reduce pneumococcal disease burden in adults aged 50–64 years, (2) age-based recommendations would be easier to implement than risk-based recommendations, and (3) might increase opportunities to vaccinate adults before individuals developed health conditions increasing their risk for pneumococcal disease. Despite these deliberations, age-based PCV20 use at the age of 50 years was rejected by ACIP at that point [2]. However, in 2024, new evidence for consideration of age-based vaccination for those 50–64 years of age resulted in recommendations for routine vaccination at age 50 years [12]. 

This study was built on the Markov model incorporating the infectious disease modeling components that incorporate background adult pneumococcal illness and vaccination rates, epidemiological changes of IPD and NBP due to routine childhood pneumococcal vaccination, and protective effectiveness over time. Emerging trends that indicate limited indirect benefit in adults aged 50–64 years old and high disease burden from *Streptococcus pneumoniae* in this age group emphasizes the need for direct vaccination in adults [2, 3]. We incorporated the latest estimates of vaccine efficacy used by ACIP in their models for PCV20, and PCV15 followed by PPSV23 [18]. While we used race-based treatment costs and risk-factor data for the Black population compared with non-Black adults, treatment costs are likely to be higher for harder-to-reach individuals impacted by structural racism [8]. Furthermore, we unable to include sub-group analyses on Hispanic/Latinx populations due to lack of IPD and NBP risk profiles for Hispanic Americans within Active Bacterial Surveillance data [21]. In addition, we considered strategies for increasing vaccine uptake, and its impact on illness in the Black, non-Black, and overall population. This study complements the investigation led by Wateska et al. (2022) and provides the US payers with financial implications of adopting an alternative scenario given available resources and budget constraints.

Our study on adult pneumococcal vaccination programs offers insights relevant to high-income and low- and middle-income countries (LMICs). While the U.S. context provides a high-income setting scenario, LMICs face unique challenges in implementing such programs, including limited healthcare budgets, healthcare infrastructure, and different epidemiological patterns. Thindwa et al. noted the optimal vaccination strategies may vary significantly between countries, driven by population demographics, disease incidence, and vaccine effectiveness [22]. Our findings highlight the potential for adult pneumococcal vaccination to reduce disease burden but emphasize the critical need for context-specific approaches that consider local healthcare infrastructure and economic constraints. Future research should focus on generating more nuanced, region-specific epidemiological and economic burden data to guide evidence-based vaccination strategies in diverse global settings.

We limited our analyses to a time horizon of 3 years, which is not long enough to forecast public health benefits. With the switch from PCV13 to PCV15 in the routine infant vaccination program, changes in the epidemiology of IPD are anticipated. As noticed worldwide, serotype replacement occurs around 3 years after introducing a new pneumococcal program leading to a reduction of public health benefits and requiring an evaluation of vaccine programs [23]. Due to this the PCV13 adult vaccination program in the US was discontinued due to limited benefits [24]. Therefore, after 3 years of an adult pneumococcal vaccination program for those aged 50–64-year-olds, it needs to be assessed in the context of the recent epidemiological changes. We used vaccine conservative cost estimates from the CDC vaccine price list and conducted sensitivity analyses around +/-50% of the cost per vaccine dose [25]. However, if a two-dose series of PCV15 and PPSV23 is priced competitively, incorporating administration fees, it is likely to provide broad serotype coverage through vaccination. We did not consider a newly approved adult-specific vaccine (i.e. PCV21) that could address pneumococcal disease in adults [26]. As such, this analysis does not reflect budget impact of including PCV21 vaccine in the adult vaccination program,. In this study, we did not consider the potential impact of the COVID-19 pandemic on IPD cases.

## Conclusions

Despite ACIP (2023) recommendations that adults under 65 years old with risk factors receive either PCV20 or PCV15 (the latter followed one year later by PPSV23), substantial pneumococcal disease burden remained, particularly in healthy adults aged 50–64 years who could likely benefit from vaccination. Budgets ranging from $6 billion for PCV20 alone to $9 billion for PCV15/PPSV23 programs are associated with routine adult pneumococcal vaccination for those aged under 65 years old over 3 years. Given higher pneumococcal disease burden, a routine adult pneumococcal program with one-dose strategy for all Black adults aged 50–64 years would have considerable health and economic benefits with the $850 million increase in budget.

## Electronic supplementary material

Below is the link to the electronic supplementary material.

Supplementary Material 1

## Data Availability

Data is provided within the manuscript or supplementary information files.

## Footnotes

1 Serotypes 1, 3, 4, 5, 6 A, 6B, 7 F, 9 V, 14, 18 C, 19 A, 19 F, 22 F, 23 F, and 33 F.

2 Serotypes 1, 3, 4, 5, 6 A, 6B, 7 F, 8, 9 V, 10 A, 11 A,12 F, 14,15B, 18 C, 19 A, 19 F, 22 F, 23 F, and 33 F.
