# Supplementary material for: A budget impact analysis of 15- or 20- valent pneumococcal conjugate vaccine use in all US adults aged 50–64 years old compared to those with high-risk conditions from US payer perspective
Source: BMC Public Health. 2025 Jun 2;25:2042. doi: 10.1186/s12889-025-22827-9 (PMC12128259; doi:10.1186/s12889-025-22827-9)
Supplement: Supplementary file 1 — Supplementary Material 1 [file 12889_2025_22827_MOESM1_ESM.docx]

**Supplementary**

**Title:** A Budget Impact Analysis of 15- or 20-valent pneumococcal conjugate vaccine use in all US adults aged 50-64 years old compared to those with high-risk conditions

**Sub-heading:** Adult Pneumococcal Program BIA

Table of Contents

[eFigure1: Comparison of Vaccine Eligible American Adults Aged 50-64 Years Under Different Scenarios 2](#_Toc188897447)

[eTable1: Market Share by Reference and Adoption Case 3](#_Toc188897448)

[eTable2: Adults Aged 50-64 Years Eligible for Vaccines by Reference and Adoption Case 3](#_Toc188897449)

[eTable3: Budget impact per year by Current Environment, New Environment, and Net Impact for PCV20 alone 3](#_Toc188897450)

[eTable4: Budget impact per year by Current Environment, New Environment, and Net Impact for PCV15 followed by PPSV23 4](#_Toc188897451)

## eFigure1: Comparison of Vaccine Eligible American Adults Aged 50-64 Years Under Different Scenarios


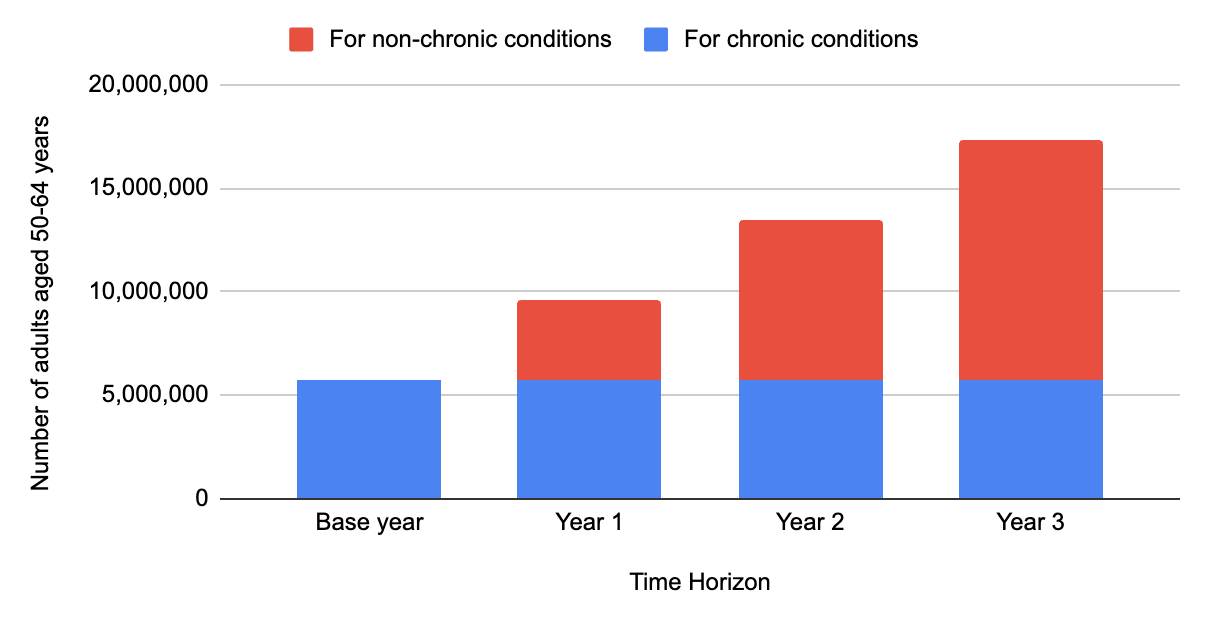


## eTable1: Market Share by Reference and Adoption Case

|  | Reference Case | | | | Adoption Case | | | |
| --- | --- | --- | --- | --- | --- | --- | --- | --- |
|  | **Base year** | **Year 1** | **Year 2** | **Year 3** | **Base year** | **Year 1** | **Year 2** | **Year 3** |
| For chronic conditions | 23% | 23% | 23% | 23% | 23% | 23% | 23% | 23% |
| For non-chronic conditions | 0% | 0% | 0% | 0% | 0% | 10% | 20% | 30% |
| Total | **23%** | **23%** | **23%** | **23%** | **23%** | **33%** | **43%** | **53%** |

## eTable2: Adults Aged 50-64 Years Eligible for Vaccines by Reference and Adoption Case

|  | Reference Case | | | | Adoption Case | | | |
| --- | --- | --- | --- | --- | --- | --- | --- | --- |
|  | **Base year** | **Year 1** | **Year 2** | **Year 3** | **Base year** | **Year 1** | **Year 2** | **Year 3** |
| For chronic conditions | 5,731,048 | 5,731,048 | 5,731,048 | 5,731,048 | 5,731,048 | 5,731,048 | 5,731,048 | 5,731,048 |
| For non-chronic conditions | 0 | 0 | 0 | 0 | 0 | 3,867,669 | 7,735,339 | 11,603,008 |
| Total | **5,731,048** | **5,731,048** | **5,731,048** | **5,731,048** | **5,731,048** | **9,598,718** | **13,466,387** | **17,334,057** |

## eTable3: Budget impact per year by Current Environment, New Environment, and Net Impact for PCV20 alone

|  | **Base year** | **Year 1** | **Year 2** | **Year 3** | **Total** |
| --- | --- | --- | --- | --- | --- |
| Current environment | | | | | |
| PCV20 (for chronic conditions) | $1,573,622,841 | $1,573,622,841 | $1,573,622,841 | $1,573,622,841 | $1,573,622,841 |
| PCV20 (for non-chronic conditions) | $0 | $0 | $0 | $0 | $0 |
| **Total** | **$1,573,622,841** | **$1,573,622,841** | **$1,573,622,841** | **$1,573,622,841** | **$6,294,491,363** |
| New environment | | | | | |
| PCV20 (for chronic conditions) | $1,573,622,841 | $1,573,622,841 | $1,573,622,841 | $1,573,622,841 | $6,294,491,363 |
| PCV20 (for non-chronic conditions) | $0 | $1,075,830,942 | $2,151,661,884 | $3,227,492,826 | $6,454,985,653 |
| **Total** | **$1,573,622,841** | **$2,649,453,783** | **$3,725,284,725** | **$4,801,115,667** | **$12,749,477,015** |
| **Net Budget Impact** | | | | | |
| PCV20 (for chronic conditions) | $0 | $0 | $0 | $0 | $0 |
| PCV20 (for non-chronic conditions) | $0 | $1,075,830,942 | $2,151,661,884 | $3,227,492,826 | $6,454,985,653 |
| **Total** | **$0** | **$1,075,830,942** | **$2,151,661,884** | **$3,227,492,826** | **$6,454,985,653** |

## eTable4: Budget impact per year by Current Environment, New Environment, and Net Impact for PCV15 followed by PPSV23

|  | **Base year** | **Year 1** | **Year 2** | **Year 3** | **Total** |
| --- | --- | --- | --- | --- | --- |
| Current environment | | | | | |
| PCV15/PPSV23 (for chronic conditions) | $2,214,759,872 | $2,214,759,872 | $2,214,759,872 | $2,214,759,872 | $8,859,039,487 |
| PCV15/PPSV23 (for non-chronic conditions) | $0 | $0 | $0 | $0 | $0 |
| **Total** | **$2,214,759,872** | **$2,214,759,872** | **$2,214,759,872** | **$2,214,759,872** | **$8,859,039,487** |
| New environment | | | | | |
| PCV15/PPSV23 (for chronic conditions) | $2,214,759,872 | $2,214,759,872 | $2,214,759,872 | $2,214,759,872 | $8,859,039,487 |
| PCV15/PPSV23 (for non-chronic conditions) | $0 | $1,514,153,924 | $3,028,307,848 | $4,542,461,772 | $9,084,923,545 |
| **Total** | **$2,214,759,872** | **$3,728,913,796** | **$5,243,067,720** | **$6,757,221,644** | **$17,943,963,031** |
| **Net Budget Impact** | | | | | |
| PCV15/PPSV23 (for chronic conditions) | $0 | $0 | $0 | $0 | $0 |
| PCV15/PPSV23 (for non-chronic conditions) | $0 | $1,514,153,924 | $3,028,307,848 | $4,542,461,772 | $9,084,923,545 |
| **Total** | **$0** | **$1,514,153,924** | **$3,028,307,848** | **$4,542,461,772** | **$9,084,923,545** |
